# Microbiota, Immune System and Autism Spectrum Disorders: An Integrative Model Towards Novel Treatment Options

**DOI:** 10.1192/j.eurpsy.2022.206

**Published:** 2022-09-01

**Authors:** D. Marazziti, B. Carpita, S. Palermo, E. Parra, L. Dell’Osso

**Affiliations:** 1 University of Siena, Department Of Chemistry- Biotechnology And Pharmacology, Siena, Italy; 2 University of Pisa, Department Of Clinica Amdf Experimental Medicine, Section Of Psychiatry, Pisa, Italy; 3 University of Pisa, Section of Psychiatry, Clinical And Experimental Medicine, Pisa, Italy; 4 University of Pisa, Department Of Clinical And Experimental Medicine, Pisa, Italy; 5 University of Pisa, Dept. Of Clinical And Experimental Medicine, Pisa, Italy

## Abstract

The functioning of the central nervous system (CNS) is the result of the integration of bidirectional messages between the brain and peripheral organs. Despite the anatomical separation, gut microbiota, i.e., the microorganisms colonising the gastrointestinal tract, is related to the CNS through the so-called “gut–brain axis” that is also involved in immune processes. The recent literature indicates that the gut microbiota may affect brain functions through endocrine and metabolic pathways, antibody production and the enteric network, while supporting its possible role in the onset and maintenance of several neuropsychiatric and neurodevelopmental disorders, such as autism spectrum disorders (ASDs). The term ASDs includes autistic disorder, Asperger’s syndrome, childhood disintegrative and pervasive developmental disorders not otherwise specified different. All these conditions are characterised by persistent deficits in social communication and social interaction, as well as limited and repetitive behaviours, interests or activities. In the last two decades, an impressive number of cross-sectional studies reported significant differences in microbiota composition between children with an ASD and controls, thus strengthening the hypothesis of a possible link between GI dysbiosis and ASD. The amount of studies documenting the possible involvement of microbiota in ASD pathogenesis led to considering whether treatments acting on gut flora could ameliorate ASD symptoms. The available findings, although preliminary, would indicate data gut microbiota might represent an interesting field of research for a better understanding of the pathophysiology of ASD (of also of other neuropsychiatric disorders), and possibly a target for the development of innovative treatments just labelled as “psychobiotics”.

**Disclosure:**

No significant relationships.

